# YOLOv8-Seg: a deep learning approach for accurate classification of osteoporotic vertebral fractures

**DOI:** 10.3389/fradi.2025.1651798

**Published:** 2025-10-14

**Authors:** Feng Yang, Yuchen Qian, Heting Xiao, Zhiheng Gao, Xuewen Zhao, Yuwei Chen, Haifu Sun, Yonggang Li, Yu Wang, Lingjie Wang, Yusen Qiao, Tonglei Chen

**Affiliations:** ^1^ Department of Orthopedics, Suzhou Ninth People’s Hospital, Suzhou, Jiangsu, China; ^2^ Department of Orthopedics, The First Affiliated Hospital of Soochow University, Suzhou, Jiangsu, China

**Keywords:** deep learning, osteoporotic vertebral fracture, YOLOv8-Seg, computed tomography (CT), fracture classification

## Abstract

**Introduction:**

This study investigates the application of a deep learning model, YOLOv8-Seg, for the automated classification of osteoporotic vertebral fractures (OVFs) from computed tomography (CT) images.

**Methods:**

A dataset of 673 CT images from patients admitted between March 2013 and May 2023 was collected and classified according to the European Vertebral Osteoporosis Study Group (EVOSG) system. Of these, 643 images were used for training and validation, while a separate set of 30 images was reserved for testing.

**Results:**

The model achieved a mean Average Precision (mAP50-95) of 85.9% in classifying fractures into crush, anterior wedge, and biconcave types.

**Discussion:**

The results demonstrate the high proficiency of the YOLOv8-Seg model in identifying OVFs, indicating its potential as a decision-support tool to streamline the current manual diagnostic process. This work underscores the significant potential of deep learning to assist medical professionals in achieving early and precise diagnoses, thereby improving patient outcomes.

## 1 Introduction

Osteoporotic vertebral fractures (OVFs) are often caused by minor external forces, typically resulting in mild compression fractures of the vertebral body. In severe cases of osteoporosis or due to inappropriate treatment, these fractures can escalate into vertebral body burst fractures, significant collapse, and kyphosis (1–3). OVFs not only trigger symptoms of spinal cord nerve damage but also adversely affect cardiopulmonary and gastrointestinal functions, thereby increasing mortality risk. Such conditions place substantial burdens on individuals, families, and society at large (4–6). Therefore, thorough investigations into the prevention and treatment of OVFs are crucial to improve patient survival quality and reduce mortality rates (7, 8).

Imaging serves as the principal method for diagnosing orthopedic conditions, including fractures, osteoarthritis, bone tumors, etc. (9). Misdiagnosis, often due to image misinterpretation or misjudgment, is prevalent in clinical settings. This issue is frequently linked to the inexperience or heavy workload of the radiologist, compounded by the subtle or atypical nature of the lesions (10). Addressing misdiagnoses in orthopedic diseases is vital, as incorrect diagnoses can severely impact patient outcomes. For example, a misdiagnosed fracture could delay surgical intervention, leading to complications like malunion or osteoarthritis. Similarly, a delayed diagnosis of a bone tumor could prevent timely surgical intervention, resulting in exacerbated symptoms and reduced functionality (11). From a clinical perspective, developing a user-friendly diagnostic model that facilitates early and accurate medical image diagnosis by less experienced physicians is essential. The integration of deep learning technology in clinical settings primarily aims at the swift identification of abnormal structures or regions within medical images, providing critical reference points for physicians' assessments and diagnoses (12–16). This study introduces a novel fracture classification method leveraging YOLOv8-Seg technology, aimed at refining the complex manual diagnostic processes.

## 2 Methods

### 2.1 Study subjects

The dataset comprised 643 computed tomography (CT) images of osteoporotic vertebral fractures collected from our hospital and affiliated institutions between March 2013 and May 2023. These images, which originated from patients treated for osteoporotic vertebral fractures, distinctly revealed the fractures.

### 2.2 YOLOv8-based fracture classification

This research introduces a novel fracture classification method utilizing the YOLOv8 deep learning network. YOLOv8 has been employed to analyze a substantial dataset of pre-labeled CT images representing various types of fractures. The network extracts distinctive features from these samples, which serve as inputs for the classifier, thereby facilitating accurate classification of the test data corresponding to different fracture types. Besides, we used the official Ultralytics YOLOv8-Seg implementation (version 8.0.*),specified the initial learning rate (lr0 = 0.01), final learning rate (lrf = 0.01), batch size (batch = 16), and number of training epochs (epochs = 300),detailed the techniques used [e.g., Mosaic (enabled for first 90% of epochs), horizontal flipping, scaling (±10%), and rotations (±10 degrees)] as part of the model's built-in augmentation pipeline and used the AdamW optimizer with a weight decay of 5 × 10^−5^.

### 2.3 Data preprocessing

In preparation for input into YOLOv8, the CT images undergo a preprocessing stage. This stage involves standardizing the images to a uniform size and resolution and converting them to grayscale. The conversion to grayscale, which reduces the number of image channels, enhances the efficiency of image processing. LABELME, a Python-based tool, is utilized for implementing this grayscale processing (17).

### 2.4 Dataset construction

Under the supervision of an experienced orthopedic surgeon, a medical trainee classified and confirmed CT images using the Imaging Labeling system, thus constructing the training and validation sets for the deep learning networks. This process followed data preprocessing guidelines. According to the 1999 EVOSG OVF classification, vertebral fractures are categorized as crush (complete collapse of the vertebral body), anterior wedge (collapse of the anterior border of the vertebral body), and biconcave (collapse of the central portion of the body) (6). For this study, 643 osteoporotic vertebral fracture images were selected for training, including 120 crush, 198 anterior wedge, and 325 biconcave types. Additionally, 30 images were chosen as the test sample, with 10 of each fracture type. Though the proportion of samples of the three types of fractures was seriously unbalanced, we have employed class-weighted loss functions to mitigate this issue, increasing the loss penalty for misclassifications on the minority classes.

### 2.5 Applying YOLOv8 to extract features

**Figure 1:**
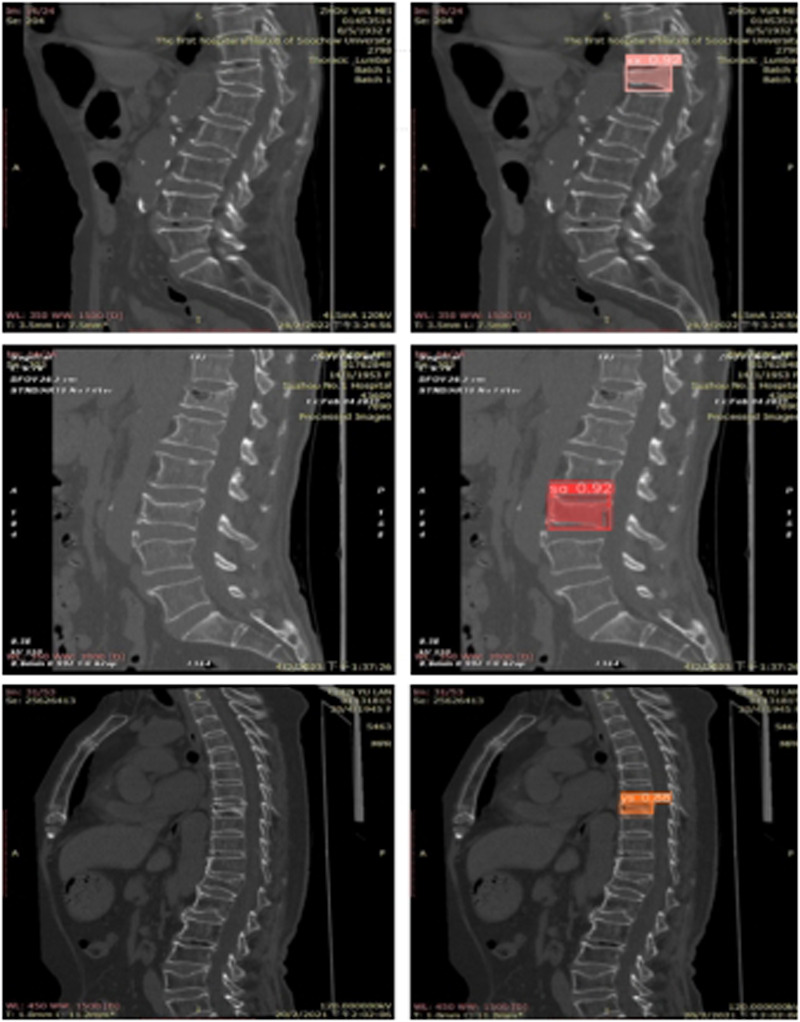
The YOLOv8 architecture.

YOLOv8, the latest iteration of the YOLO object detection model (18, 19), retains the architecture of its predecessors but incorporates several enhancements, such as a new neural network architecture that integrates both the Feature Pyramid Network (FPN) and the Path Aggregation Network (PAN), along with a new labeling tool that streamlines the annotation process (18, 19). The FPN progressively decreases the spatial resolution of the input image while augmenting the number of feature channels, thereby generating feature maps capable of detecting objects across various scales and resolutions. The PAN architecture amalgamates features from different levels of the network through skip connections, enabling the network to capture features at multiple scales and resolutions effectively. This capability is crucial for accurately detecting objects of varying sizes and shapes. Figure 1 shows the architecture of YOLOv8.

### 2.6 Evaluation indicators

In this study, the evaluation of the overall classification effect was conducted using mean average precision (mAP) values derived from the test set. mAP, a widely used metric for object detection, calculates the average precision (AP) across all classes at predetermined Intersection over Union (IoU) thresholds. Precision is determined based on true positives and false positives in object detection. A prediction is deemed a true positive if the IoU between the predicted box and the ground truth exceeds the set IoU threshold, while it is considered a false positive if the IoU falls below this threshold. The mean average precision (mAP) for each class is calculated by iterating over a series of thresholds, starting from an IoU of 0.5 and increasing in steps of 0.05 up to 0.95. The class AP represents the average precision across this range. By computing this value for all classes and averaging the results, the mAP50-95 is obtained.

Formula 1. Recalls (*n*) = 0, Precisions (*n*) = 1, *n* = Number of thresholds

(1) $$\mathrm{AP}=\sum_{k=0}^{k=n-1}\left[\mathrm{Recalls}(k)-\mathrm{Recalls}(k+1)]\;\times \mathrm{Precisions}(k\right)$$

Formula 2. AP*_k_* = the AP of class *k*, *n* = the number of classes

(2) $$\mathrm{mAP}=\frac{1}{n}\sum_{k=1}^{k=n}AP_{k}$$

### 2.7 Experimental environment

The experimental setup employed an NVIDIA A6000 graphics card alongside the YOLOv8n-seg model, which is designed for simultaneous target detection and instance segmentation. This dual-function capability allows the model to perform both tasks concurrently. This enables the model to complete two tasks concurrently.

## 3 Results

For clarity in the labeling process, this study adopts specific nomenclature: “ys” for crush fractures, “xx” for wedge fractures, and “sa” for biconcave fractures. This nomenclature is consistently used in the figures.

**Figure 2:**
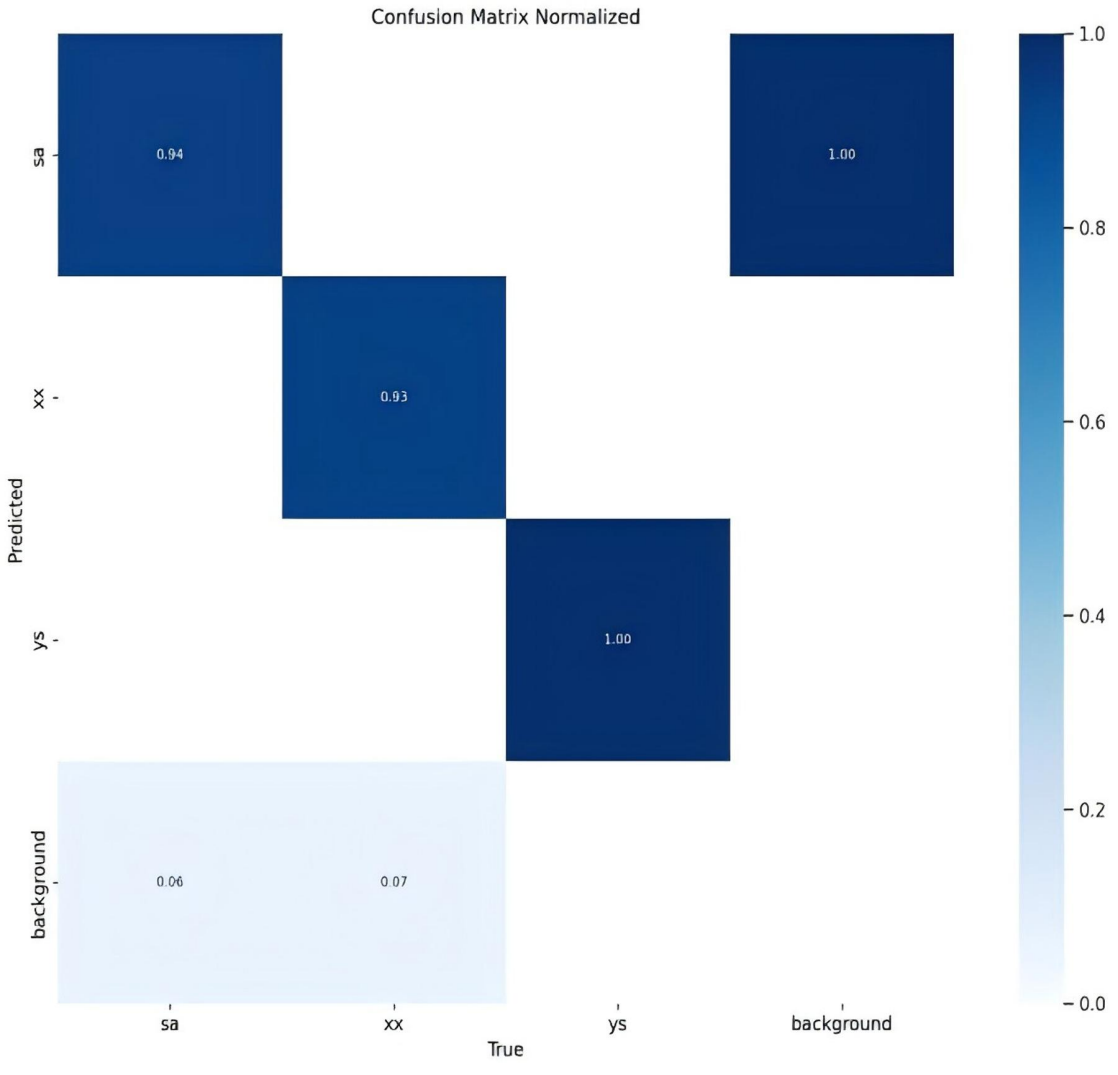
Confusion matrix normalized.

The classification results for the three types of osteoporotic vertebral fractures (OVF)—biconcave fractures, anterior wedge fractures, and crush fractures—are detailed in Figure 2 and Table 1. The YOLOv8 algorithm's performance was assessed using a comprehensive dataset. Out of the 315 biconcave fractures analyzed, 6% were not detected, while 94% were correctly identified, indicating a high level of accuracy in detecting biconcave fractures. Similarly, of the 188 anterior wedge fractures examined, 7% were not detected, and 93% were accurately identified, demonstrating the algorithm's effectiveness in recognizing anterior wedge fractures. Notably, each of the 110 crush fractures was correctly identified, resulting in a perfect detection rate of 100%. The overall mean Average Precision (mAP50-95) for the classification of these fractures was calculated to be 85.9%, reflecting the robust performance of the YOLOv8 algorithm in accurately classifying different types of osteoporotic vertebral fractures.

**Table 1:** The mean average precision (mAP50-95).

Class	All	sa	xx	ys
Images	58	58	58	58
Instances	58	32	15	11
Box (*P*)	0.943	0.937	0.926	0.965
*R*	0.957	0.938	0.933	1
mAP50	0.966	0.96	0.943	0.995
mAP50-95	0.859	0.839	0.869	0.869
Mask(P	0.943	0.937	0.926	0.965
*R*	0.957	0.938	0.933	1
mAP50	0.966	0.96	0.943	0.995
mAP50-95	0.779	0.732	0.792	0.814

**Figure 3:**
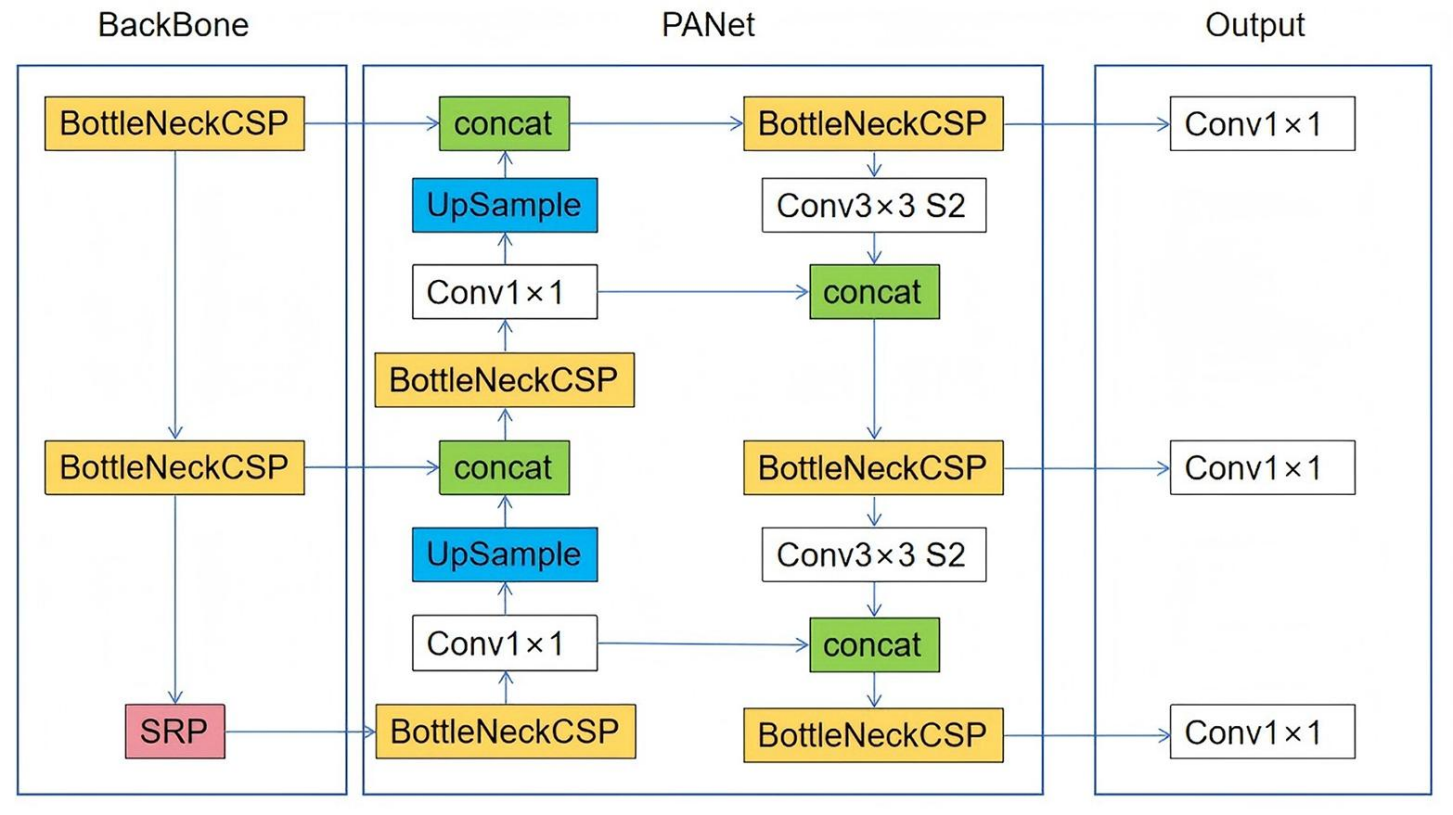
The quantity of data in the training set, the number of instances in each category, the dimensions and number of frames, the position of the center point in relation to the entire map, and the height-to-width ratio of the target in the map relative to the entire map.

Figure 3 presents data on the training set, including the number of instances in each category, the dimensions and number of frames, the position of the center point relative to the entire map, and the height-to-width ratio of the target within the map.

**Figure 4:**
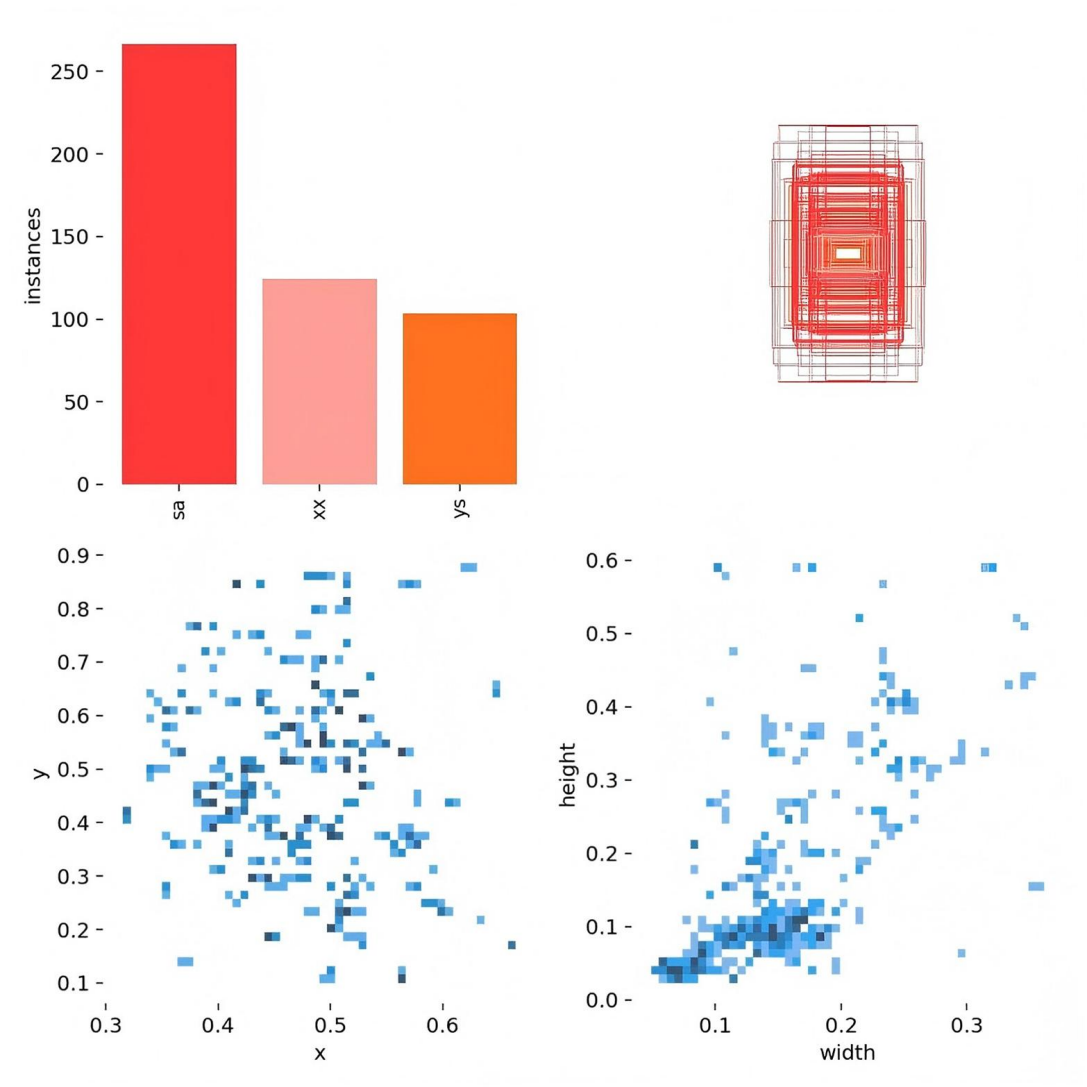
The horizontal and vertical coordinates of the center point and the height and width of the frame.

Figure 4 depicts the relationship between the horizontal and vertical coordinates of the center point and the dimensions of the frame.

**Figure 5:**
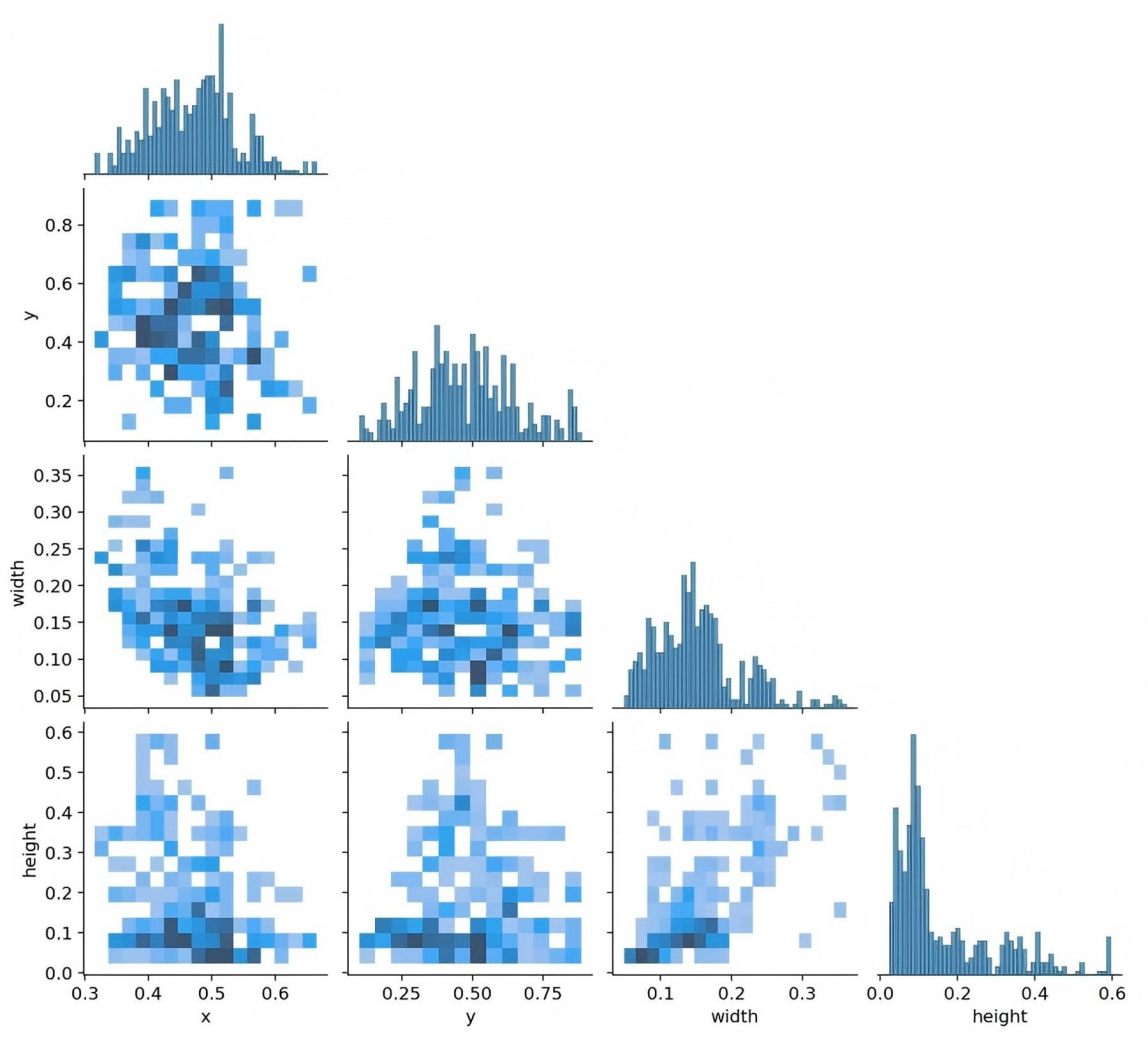
The loss functions.

Figure 5 illustrates the model's loss functions, which quantify the discrepancy between the predicted and actual values. These loss functions significantly influence the model's performance, as they guide the training process towards more accurate predictions. The positioning loss (box_loss) is represented by the error between the prediction box and the calibration box, calculated using the Generalized Intersection over Union (GIoU) metric. A lower GIoU value indicates better accuracy in positioning, reflecting a more precise overlap between the predicted and true bounding boxes. The confidence loss (obj_loss) measures the network's confidence in its predictions; a lower value suggests higher accuracy in determining the presence of an object, thus improving the model's ability to differentiate between objects and the background. Lastly, the classification loss (cls_loss) evaluates the accuracy of classifying the anchor frame relative to the calibration. A lower classification loss indicates more precise classification of detected objects into their respective categories, ensuring that the model accurately identifies the types of objects present. Collectively, these loss functions are integral to optimizing the YOLOv8 model, driving enhancements in both detection precision and classification accuracy.

**Figure 6:**
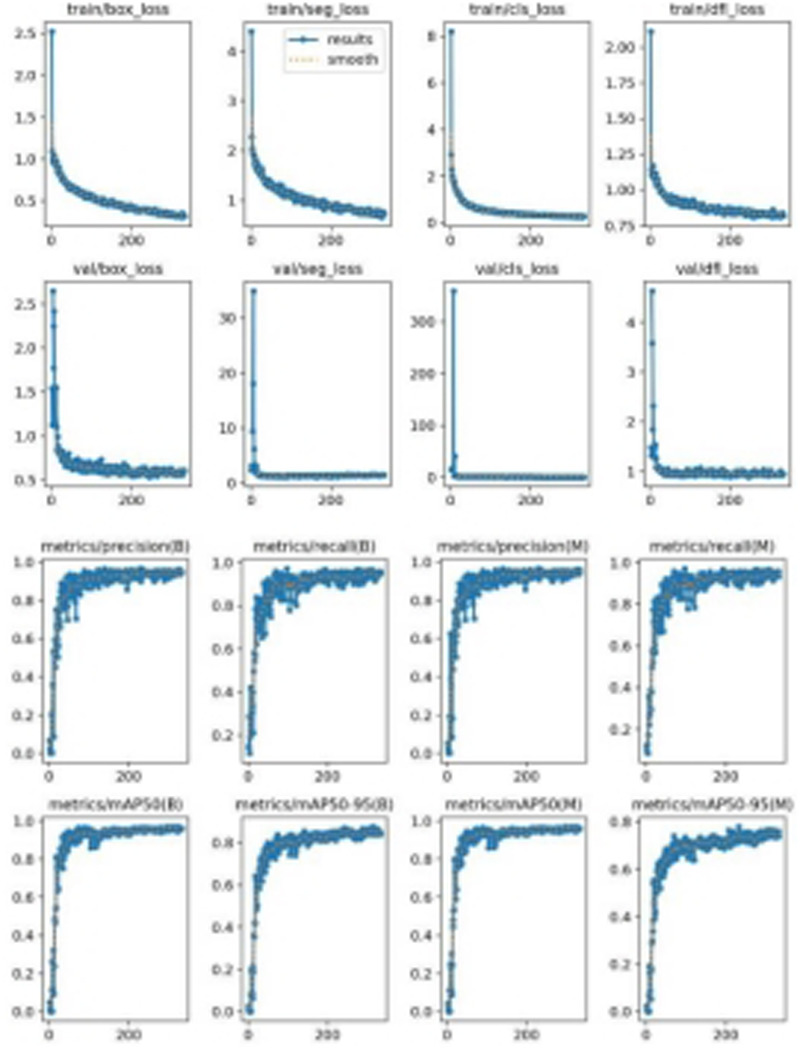
Examples of the three types classification test results.

Extensive experiments were conducted on multiple spinal computed tomography (CT) images to validate the performance of the YOLOv8 algorithm. Figure 6 illustrates some of the detection results. In this figure, regions identified as wedge fractures and biconcave fractures by the YOLOv8 algorithm are highlighted, with corresponding confidence scores annotated. Specifically, the upper row of images in Figure 6 displays the detection of wedge fractures, labeled as “xx,” while the lower row of images shows the detection of biconcave fractures, labeled as “sa.” Both types of fractures are detected with a high confidence score of 0.92.

Precision is defined as the measure of the accuracy of the model's positive predictions, calculated as the ratio of correctly identified positive instances (true positives) to all identified positive instances (both true positives and false positives). A higher precision indicates that the model commits fewer false positive errors, thereby ensuring that most of its positive predictions are accurate. Recall, in contrast, represents the proportion of actual positive samples that are correctly identified by the model, essentially measuring the model's ability to identify all relevant instances within the dataset. Specifically, recall is calculated as the ratio of true positives to the sum of true positives and false negatives. Essentially, recall quantifies the number of true positive examples in the test set that are accurately identified by the binary classifier.

The mean Average Precision (mAP) is an aggregate metric that encapsulates both precision and recall into a singular value. It is determined by the area under the precision-recall curve, where precision is plotted on the *y*-axis and recall on the *x*-axis. The mAP offers a balanced assessment of the model's performance, considering both the accuracy and completeness of the positive predictions.

**Figure 7:**
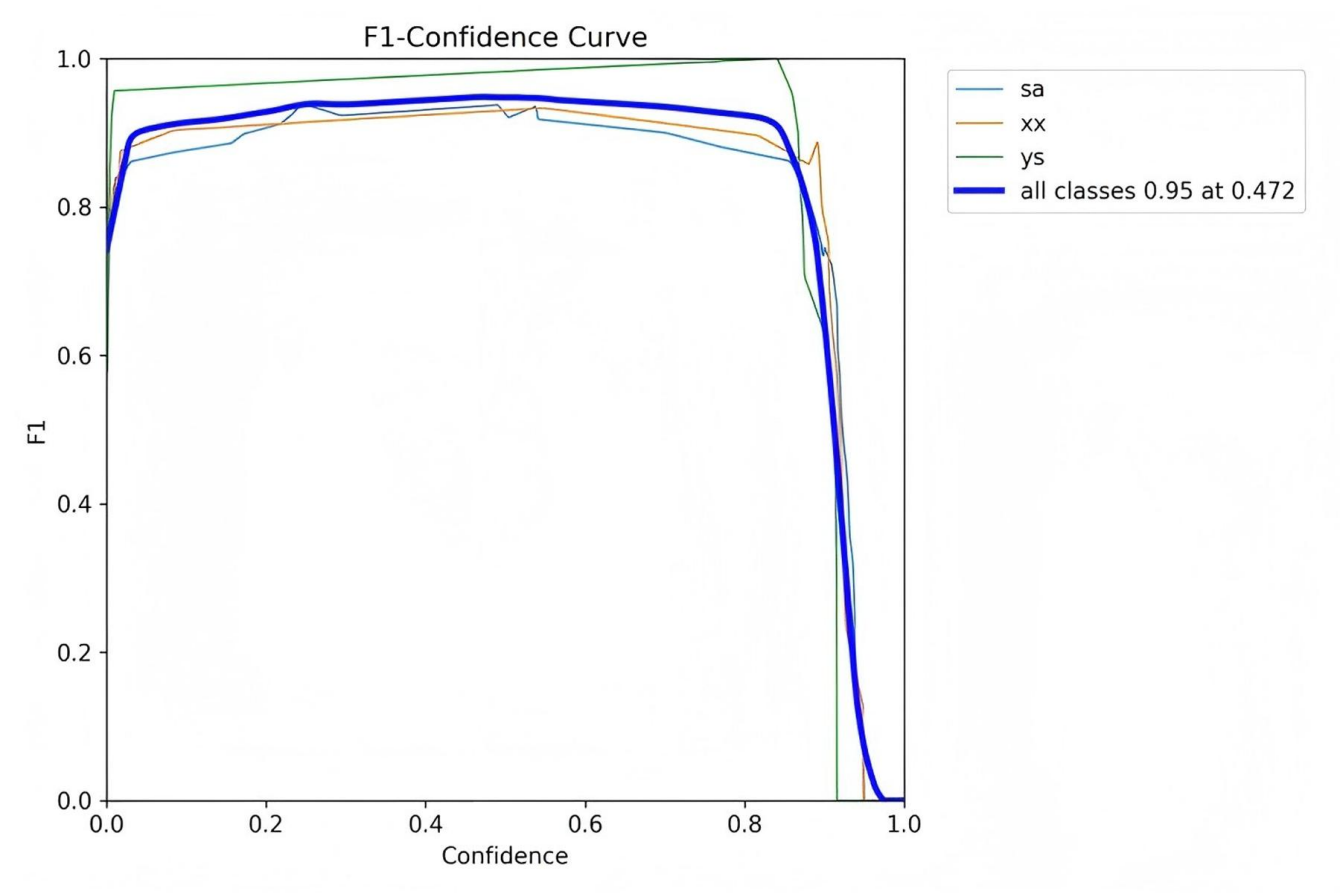
F1-confidence curve.

**Figure 8:**
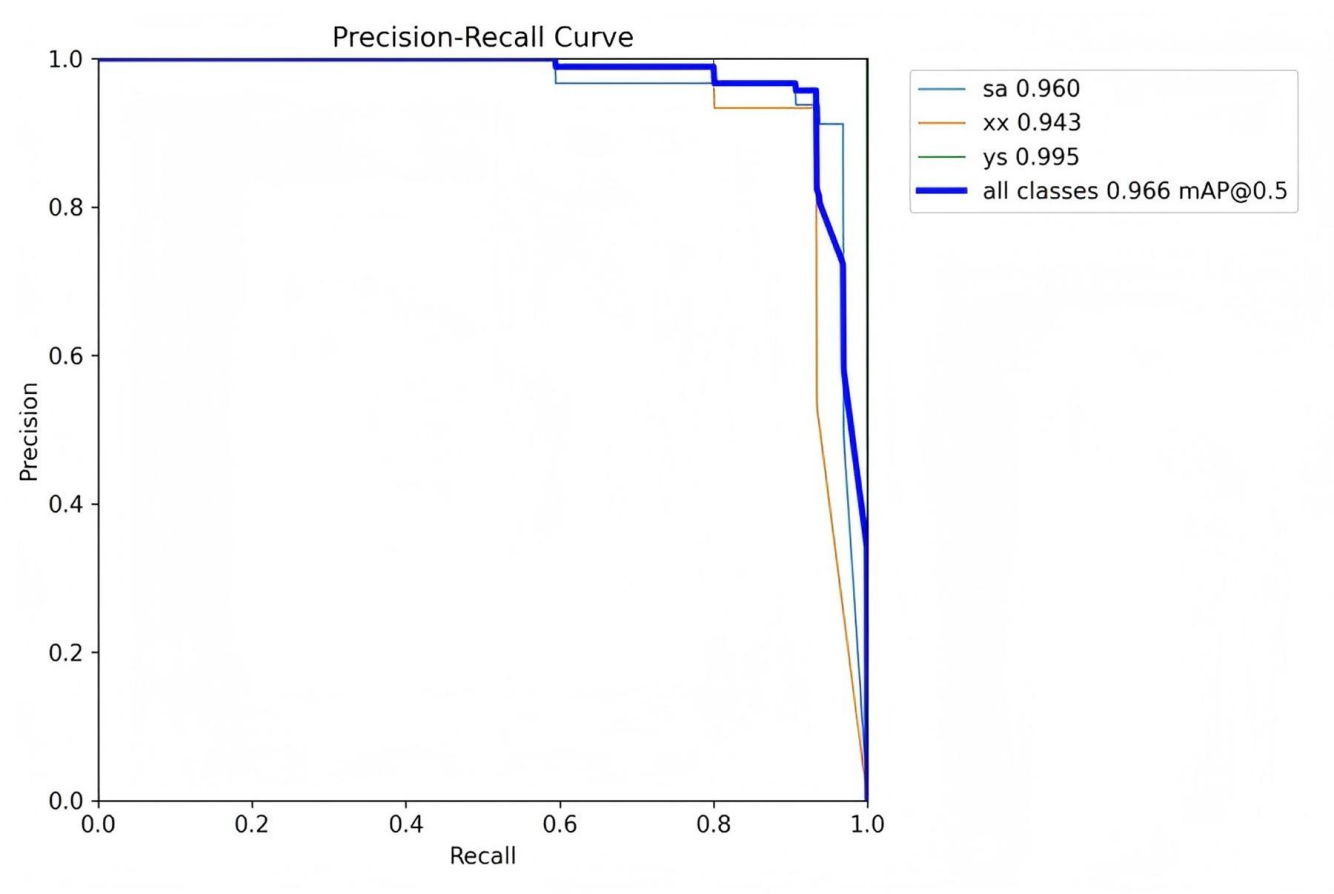
Precision-recall curve.

**Figure 9:**
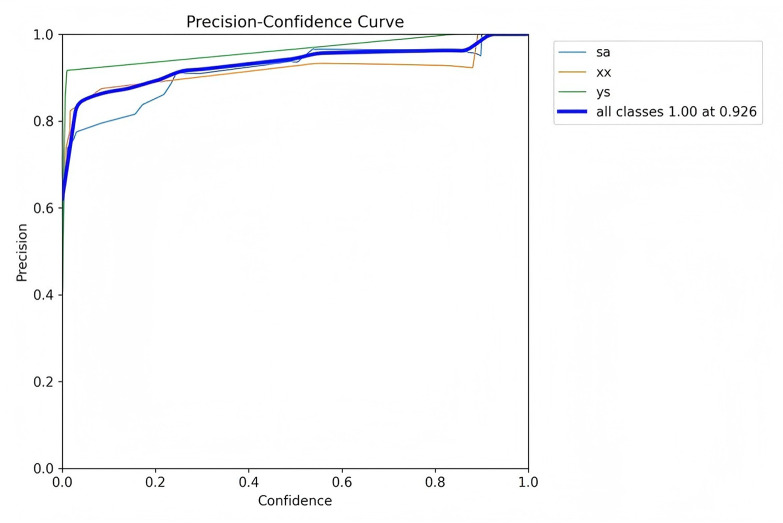
Precision-confidence curve.

**Figure 10:**
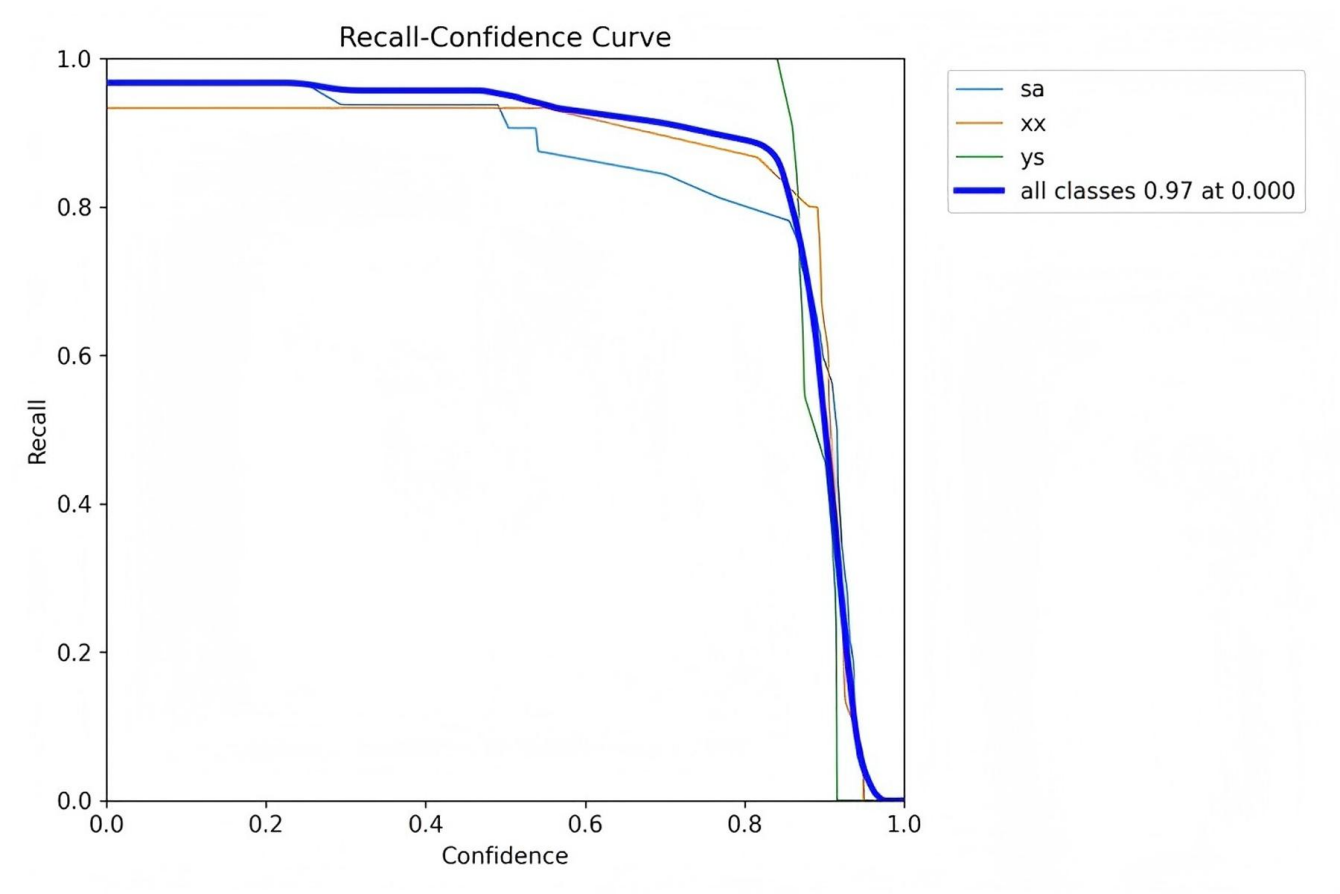
Recall-confidence curve.

Figures 7–10 depict various relationships involving the F1 score, confidence, precision, and recall. The F1 score, a harmonic mean of precision and recall, provides a single metric that balances these two aspects. These figures elucidate how alterations in the confidence threshold influence the F1 score, precision, and recall, providing insights into the trade-offs between these metrics. By examining these relationships, one can gain a deeper understanding of the model's performance and make informed decisions regarding the optimal confidence thresholds for various applications.

The YOLOv8-Seg model demonstrated superior performance in terms of mAP50-95 and inference speed against a benchmark Mask R-CNN model (with a ResNet-50 backbone) trained and tested on our dataset under identical conditions.

## 4 Discussion

This study utilized the YOLOv8-Seg deep learning model to classify osteoporotic vertebral fractures (OVFs)—specifically biconcave, wedge, and crush types—based on spinal CT images. The model achieved an overall mean Average Precision (mAP50-95) of 85.9%, demonstrating high proficiency in detecting and segmenting fracture regions. Notably, the model showed perfect detection (100%) for crush fractures, which may be attributable to their more pronounced morphological deformation compared to wedge and biconcave types.

Our results align with recent advances in applying deep learning to medical image analysis, particularly in orthopedics. For instance, Yang et al. (16) reported comparable mAP values in fracture detection using convolutional neural networks, though their model did not perform instance segmentation. The incorporation of both FPN and PAN in YOLOv8-Seg allows multi-scale feature aggregation, enhancing detection across fracture types of varying shapes and sizes—a challenge noted in earlier studies such as Zhou et al. (20). Moreover, our model's lightweight architecture offers faster inference speeds compared to heavier networks like Mask R-CNN, making it more suitable for real-time clinical applications.

The integration of YOLOv8-Seg into clinical workflows has the potential to significantly reduce diagnostic time and assist less experienced radiologists in identifying OVFs accurately. By providing automated, high-confidence fracture classifications and localizations, the system can serve as a decision-support tool, particularly in high-volume settings. Future implementation may involve embedding the model into PACS systems for real-time inference during image reading, thereby offering immediate diagnostic suggestions.

While this study demonstrates the promising performance of YOLOv8-Seg in classifying osteoporotic vertebral fractures, several limitations should be acknowledged. First, the model was trained and validated on a single-center dataset with inherent demographic and diagnostic biases, which may limit its generalizability to other populations or imaging protocols. Future studies should incorporate multi-institutional data to improve robustness and external validity. Second, the dataset exhibited significant class imbalance, particularly with underrepresentation of crush and wedge fractures. Although class-weighted loss was employed to mitigate this issue, future work could explore advanced techniques such as synthetic minority oversampling (SMOTE) or generative adversarial networks (GANs) to create more balanced training sets.

Furthermore, the current study lacks comparative validation against other state-of-the-art segmentation models or clinician performance (21–23). A future reader study involving radiologists of varying experience levels would help contextualize the model's diagnostic accuracy and practical utility. Finally, the translation of such AI tools into clinical practice faces practical barriers, including integration into existing Picture Archiving and Communication Systems (PACS), compliance with healthcare data privacy regulations, and the need for real-time inference capabilities. Future efforts should also address clinician trust and interpretability by incorporating explainable AI techniques, such as attention maps or uncertainty quantification, to provide deeper insight into model predictions.

## 5 Conclusion

This study addresses the prevalent challenges in diagnosing spinal fractures, an issue that is increasingly significant due to the aging population. It introduces cutting-edge deep learning methods for the classification of fracture diagnoses, contributing significantly to the enhancement of medical image diagnosis, assisted decision-making, and pre-operative planning for various clinical treatments. Furthermore, this research advances the precision of invasive operations and the execution of plans in areas such as surgical robotics.

It is important to note that the study's limitations include a relatively small sample size, which impacts the classification accuracy of the proposed method. Additionally, the potential for misidentification and misclassification increases with a smaller dataset. Future research will aim to overcome these limitations by developing a more robust network structure and algorithm, enhancing the accuracy of learning fracture patterns.

## Data Availability

The original contributions presented in the study are included in the article/Supplementary Material, further inquiries can be directed to the corresponding authors.
